# Supplementary material for: Knowledge-guided Contextual Gene Set Analysis Using Large Language Models
Source: ArXiv. 2025 Jun 4:arXiv:2506.04303v1. Preprint. [Version 1] (PMC12155053)
Supplement: Supplement 1 [file NIHPP2506.04303v1-supplement-1.pdf]

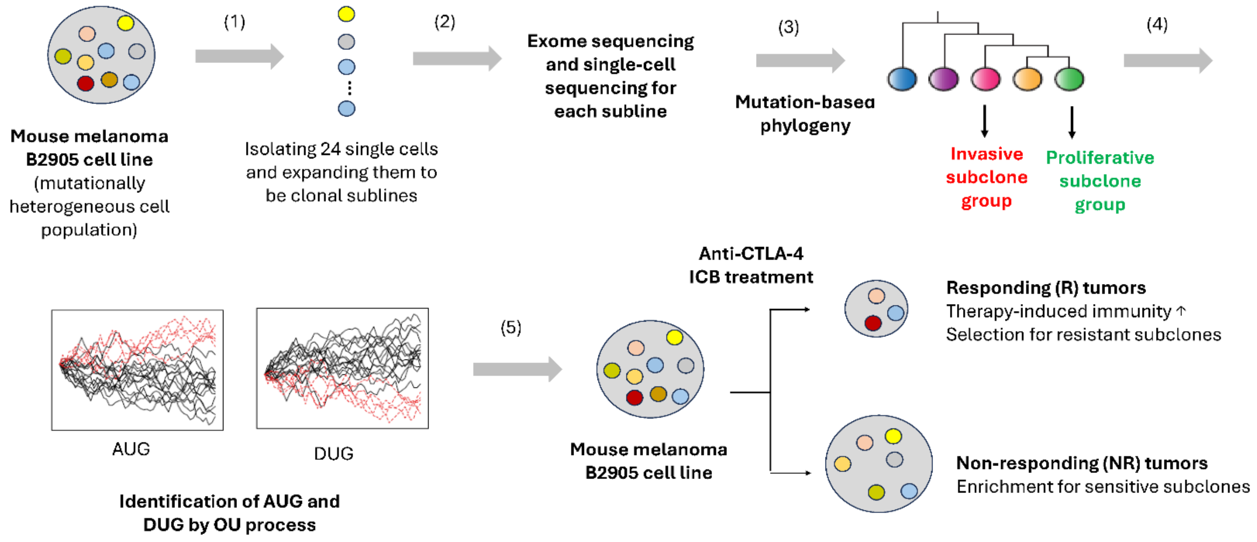

Figure S1. The pipeline proposed by Hirsch *et al.* to model evolutionary changes in gene expression of melanoma as stochastic Ornstein-Uhlenbeck (OU) processes. Briefly, 24 clonal sublines derived from the parental mouse melanoma B2905 cell line were analyzed for DNA mutations and RNA expressions. They built mutation-based phylogeny of the 24 sublines and characterized branches containing several evolutionarily related sublines, i.e. "subclone groups", whose sublines exhibited similar phenotypes. Two subclone groups were named "invasive subclone group" and "proliferative subclone group", based on their distinct phenotypes. Moreover, the former was resistant, and the latter was sensitive to immune checkpoint blockade (ICB) therapies. By analyzing evolutionary trajectories of gene expression along the phylogeny with OU process, they identified adaptively upregulated and downregulated genes (AUGs and DUGs, respectively) in the invasive (572 AUGs and 347 DUGs) and proliferative subclonal groups (169 AUGs and 1151 DUGs) (**Data file S1, Case-Study DEGs**).

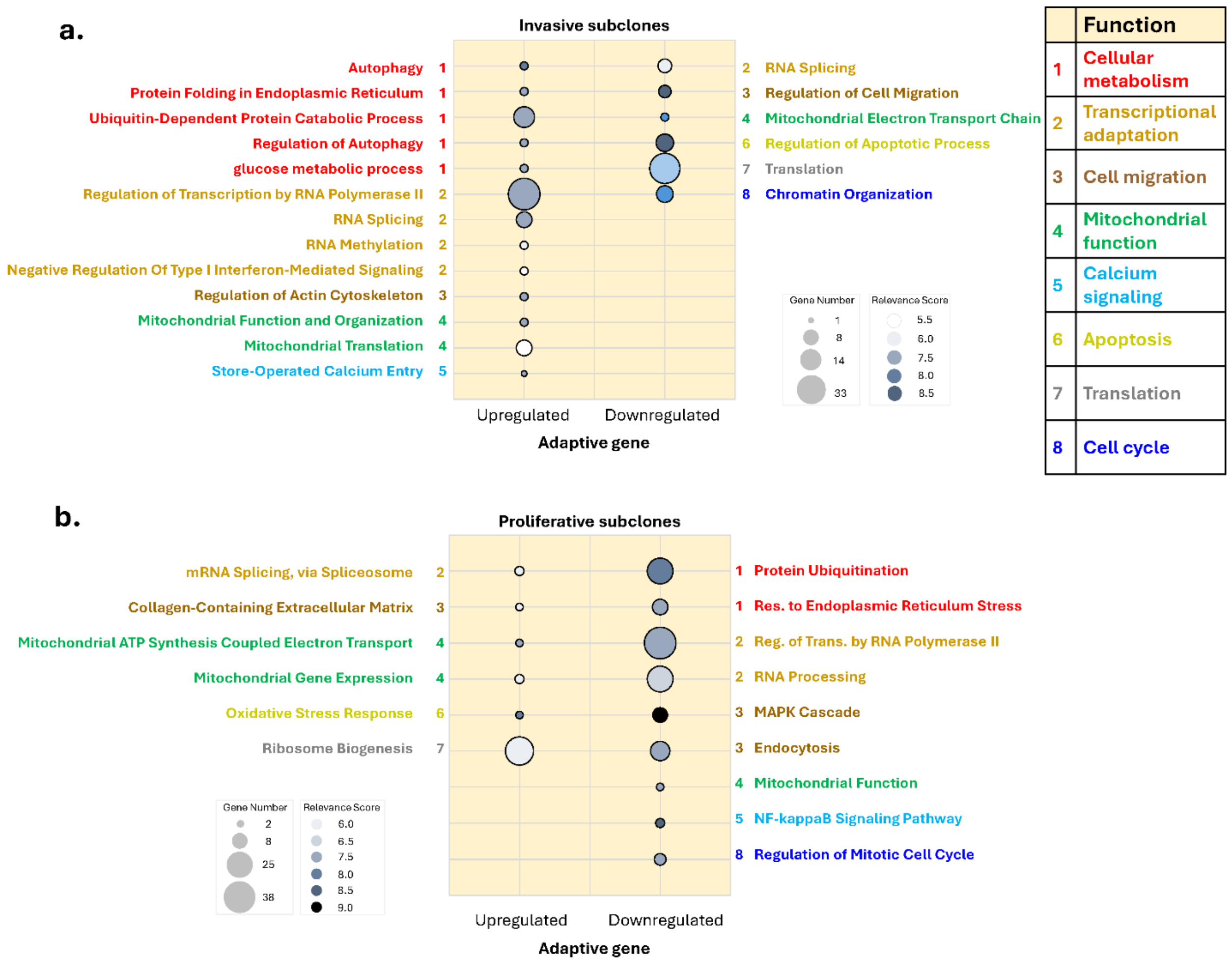

Figure S2. cGSA output pathways enriched by the adaptively upregulated genes (AUGs) and adaptively downregulated genes (DUGs) of the invasive subclone group (a) and the proliferative subclone group (b). The context is "melanoma". The results show that: (1) for the invasive subclonal group, AUGs were more enriched in category 1 (metabolism) and 2 (transcriptional adaptation) pathways with higher relevance scores. These include pathways of "Ubiquitin-dependent protein catabolic process" (14 genes, score = 7.5), "Regulation of transcription by RNA Polymerase II" (33 genes, score= 7.5), and "RNA splicing" (8 genes, score = 7.5). In contrast, DUGs of the invasive subclonal group were more enriched in category 6 (apoptosis), 7 (translation), and 8 (cell cycle) pathways with higher relevance score. These include pathways of "Regulation of apoptotic process" (10 genes, score = 8.5), "Translation" (30 genes, score = 7.0), and "Chromatin organization" (9 genes, score = 7.5). (2) for the proliferative subclonal group, AUGs were more enriched in category 6 (apoptosis) and 7 (translation) pathways with higher relevance score. These include pathways of "Oxidative stress response" (2 genes, score = 8.0), and "Ribosome biogenesis" (30 genes, score = 6.5). On the other hand, DUGs were more enriched in category 1 (metabolism), 2

(transcriptional adaptation), and 3 (cell migration) pathways with higher relevance score. These include pathways of "Protein Ubiquitination" (25 genes, score = 8.5), "Regulation of Transcription by RNA Polymerase II" (38 genes, score = 7.5), "RNA Processing" (25 genes, score = 7.0), "MAPK pathway" (8 genes, score = 9.0), and "Endocytosis" (14 genes, score = 7.5). In summary, in consistency with the observation by Hirsch *et al*, our results also demonstrate that the adaptive genes of invasive and proliferative groups show contrast functional patterns. That is, the functions referred to the adaptively up- and down-regulated genes in the invasive subclones are those to the adaptively down- and up-regulated genes in the proliferative subclones, respectively. This suggests that the two subclonal groups gained invasive and proliferative phenotypes by evolving toward two distinct transcriptional states, conferring the functions of metabolic/transcriptional adaptation and cell growth, respectively.

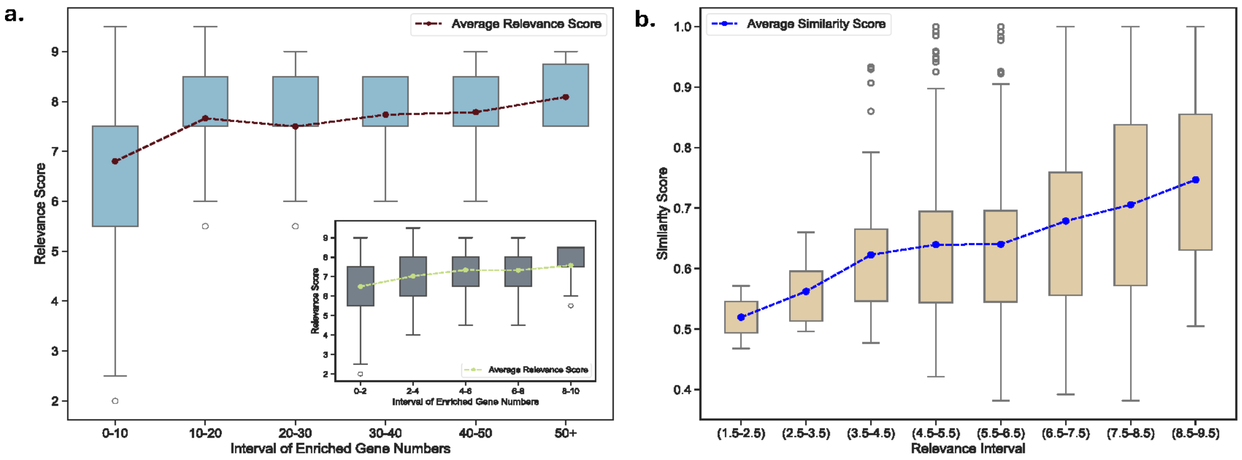

Figure S3. The results of relevance score calibration. Correlations between relevance scores generated by cGSA and the number of enriched genes of ground truths curated in databases (a), and the similarity scores calculated by MedCPT between cGSA's output and ground truths. We grouped the number of enriched genes (**a**) and relevance scores of all output pathways (**b**) into different intervals (**x-axis**). The average relevance score (**a**) and similarity score (**b**) in each interval (**y-axis**) between output pathways and their corresponding ground truths, exhibits a linear-like increasing trend. The total samples for statistical analysis are 1,469. For boxplots, the lower and upper hinges correspond to the first and third quartiles. The upper (lower) whisker extends from the hinge to the largest (smallest) value no further than (at most)1.5× IQR from the hinge (where IQR is the inter-quartile range). The middle points represent the mean values.

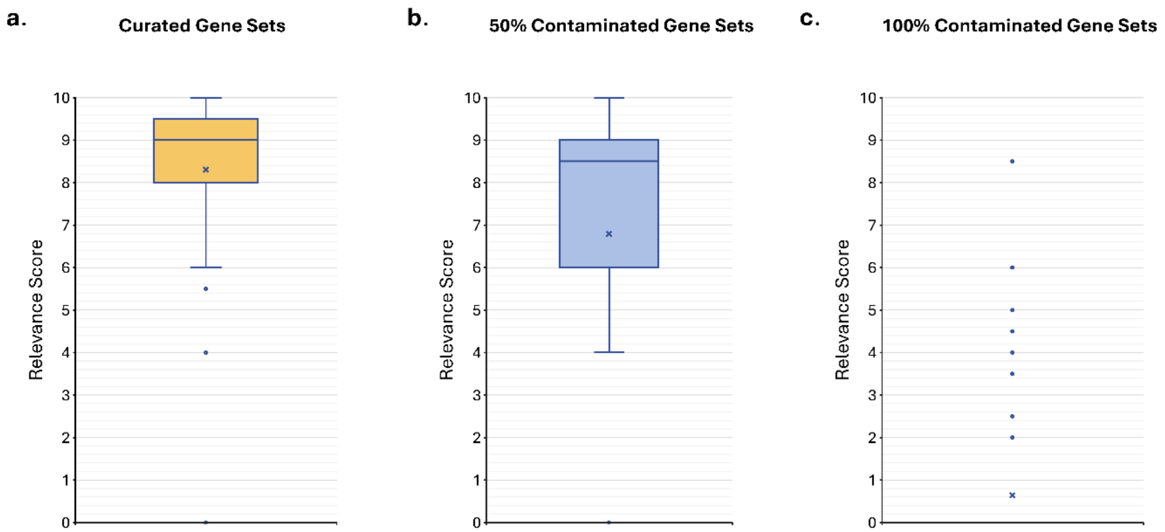

Figure S4. Relevance scores for curated gene sets and their corresponding contaminated gene sets. a, relevance scores assigned to curated gene sets containing no randomly introduced genes, with an average score of 8.31. **b**, relevance scores assigned to 50% contaminated gene sets, in which half of the genes were randomly selected from the background pool. The average score is 6.79. **c**, relevance scores assigned to 100% contaminated gene sets, where all genes were randomly selected while maintaining the same scale as curated gene sets. The average score is 0.64. The total samples for statistical analysis are 100. For boxplots, the lower and upper hinges correspond to the first and third quartiles. The upper (lower) whisker extends from the hinge to the largest (smallest) value no further than (at most)1.5× IQR from the hinge (where IQR is the inter-quartile range). The middle points represent the mean values while the middle lines represent the median values.

List of Supplementary Materials

Data file S1 (.xlsx): Differentially expressed genes (DEGs) used for evaluation and case studies.

Data file S2 (.docx): Prompts (instructions) used in the cGSA framework.

Data file S3 (.xlsx): Expert annotations for the outputs of cGSA.

Data file S4 (.xlsx): Results of two case studies.

Data file S5 (.xlsx): Evaluations of relevance scores for different DEGs.

Data file S6 (.xlsx): Example outputs obtained from the cGSA online platform.

Data file S7 (.xlsx): Raw experimental results related to Figures 2 and 4.
